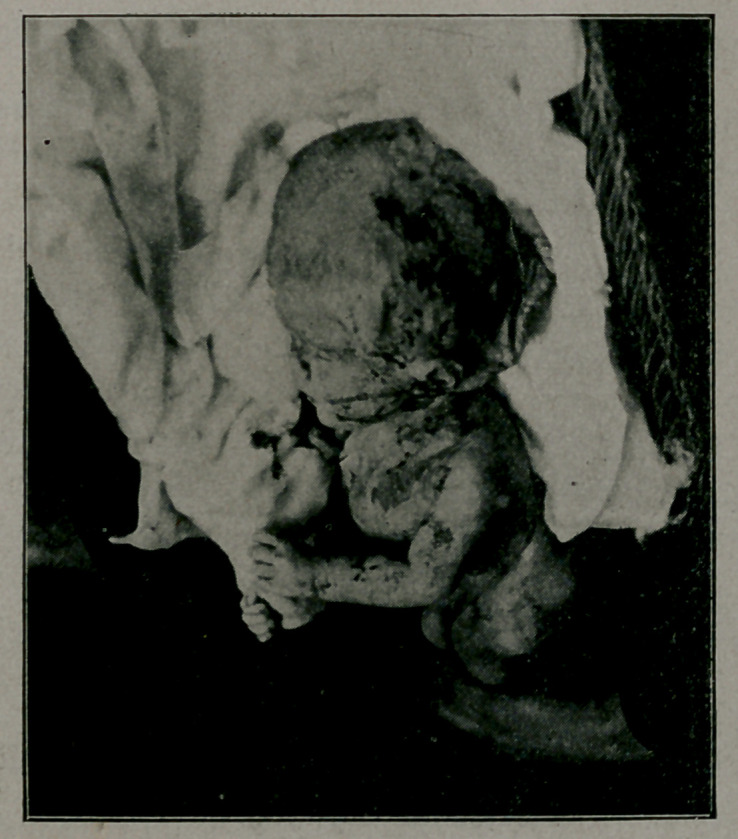# Breech Presentation—Hydrocephalus—Spina Bifida

**Published:** 1912-04

**Authors:** Frederick G. Hodgson

**Affiliations:** Atlanta, Ga.


### BREECH PRESENTATION—HYDROCEPALUS—SPINA BIFIDA.

By Frederick G. Hodgson, M. D., Atlanta, Ga.

C. P., age 24, para 1. This case was not seen until the second stage of labor. No ante partum examination had been made nor had the pelvic measurements been taken.

Labor began at 4 P. M., pains were good, the membranes rupturing at 2 a. m. Examination at this time showed the fundus to be up to the sternum. No heart sounds could be heard and the position of the child could not be outlined by abdominal palpation because of the tight uterus. Scrotum was just out of vulva. Pains were allowed to proceed for forty-five minutes, at the end of this time no progress had been made and it was decided to deliver. Under moderate chloroform anaesthesia the legs were delivered, this being extremely difficult, the left femur being fractured in so doing. Afer the delivery of the legs the child could not be pulled down at all and for this reason the arms were also difficult to deliver. Traction on the legs at this time did not bring the head down the slightest bit. Upon putting the hand into the uterus the head could be plainly felt, being about the size of an ordinary round football. Perforation was done, this being easy because of the softness of the skull bones. Upon the liberation of about three quarts of cloudy fluid the head almost dropped out.

In addition to the deformity of the head, the posterior wall of the spinal canal had failed to close for almost its entire length.

A second degree laceration of the perineum was immediately repaired and the subsequent course of the case uneventful.

424-425 Candler Bldg.

## Figures and Tables

**Figure f1:**
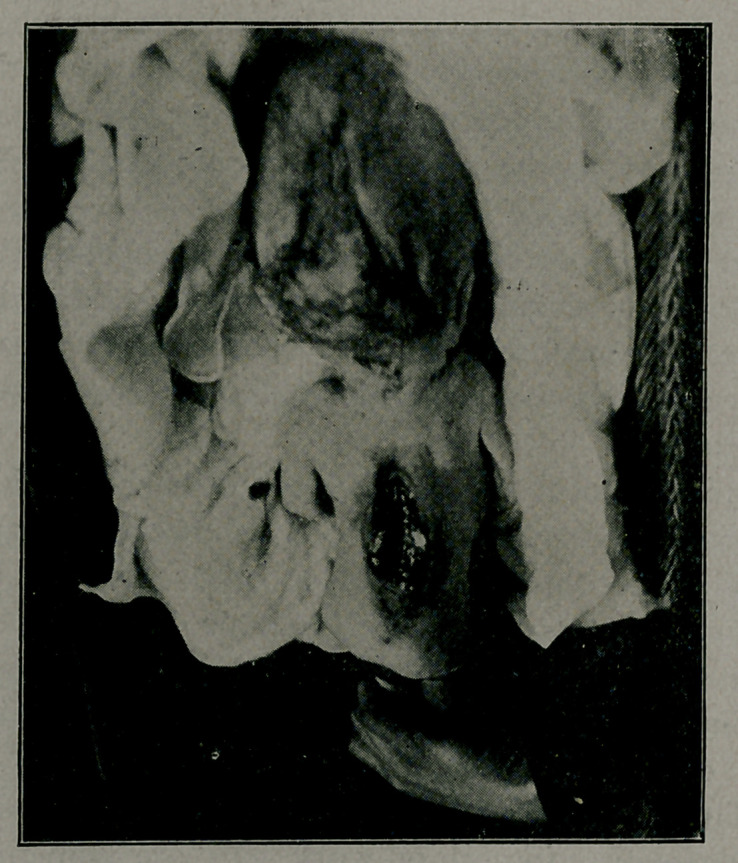


**Figure f2:**